# Construction and evaluation of a predictive model for high mental workload risk among clinical nurses: a cross-sectional study

**DOI:** 10.3389/fpubh.2026.1724229

**Published:** 2026-02-09

**Authors:** Zhongqing Yuan, Wanqing Xie, Jialin Wang, Xiaolan Li, Li Zeng, Fengxue Yang

**Affiliations:** 1 Sichuan Nursing Vocational College, Chengdu, Sichuan, China; 2 State Key Laboratory of Oral Diseases & National Center for Stomatology & National Clinical Research Center for Oral Diseases, West China Hospital of Stomatology, Sichuan University, Chengdu, Sichuan, China; 3 School of Nursing, Chengdu University of Traditional Chinese Medicine, Chengdu, Sichuan, China; 4 Sichuan Provincial Hospital of Integrated Traditional Chinese and Western Medicine, Chengdu, Sichuan, China; 5 Innovation Center of Nursing Research and Nursing Key Laboratory of Sichuan Province, West China School of Nursing, Sichuan University, Chengdu, Sichuan, China

**Keywords:** job demands-resources model, mental workload, nomogram, nurses, risk prediction

## Abstract

**Aim:**

To develop and validate a predictive model for identifying clinical nurses at high risk of mental workload (MWL) using a nomogram-based approach, grounded in the Job Demands–Resources theoretical framework.

**Methods:**

Guided by the Job Demands–Resources model, a total of 826 clinical nurses were recruited from nine tertiary hospitals. Data were collected through standardized questionnaires assessing demographic characteristics, mental workload, and psychosocial factors including emergency response capacity, perceived social support, and coping style. A total of 16 variables were screened using least absolute shrinkage and selection operator (LASSO) regression. Seven significant predictors were then entered into a binary logistic regression model and used to construct a nomogram. Model performance was assessed using the area under the curve (AUC), calibration curves, Hosmer–Lemeshow test, and 10-fold cross-validation.

**Results:**

Seven variables were identified as independent predictors of high mental workload: gender, salary satisfaction, frequency of night shifts, turnover intention, emergency response capacity, perceived social support, and negative coping style. The nomogram demonstrated good discriminative ability in both the training (AUC = 0.796, 95% CI: 0.741–0.852) and validation cohorts (AUC = 0.793, 95% CI: 0.757–0.830). Calibration curves showed strong agreement between predicted and observed outcomes. The C-index derived from bootstrap resampling was 0.761, while 10-fold cross-validation yielded a mean C-index of 0.771, indicating robust internal validity and consistent performance.

**Conclusion:**

A validated nomogram was developed to predict the risk of high mental workload among clinical nurses. The model exhibited favorable discrimination, sound calibration, and consistent internal reliability, offering an effective means for identification and focused intervention.

## 1 Introduction

As healthcare systems worldwide grapple with increasing complexity, aging populations, and chronic workforce shortages, clinical nurses are experiencing an unprecedented level of work-related stress in their professional roles. Studies reported that over 11% of clinical nurses experienced moderate to severe burnout symptoms, more than 60% worked frequent night shifts each month, and clerical responsibilities account for a noteworthy share of their workload (1–3). These factors collectively contributed to elevated mental workload (MWL) and decreased care quality. Hence, addressing the issue of high mental workload among clinical nurses is a key research area.

Mental workload (MWL) is a foundational concept from human factors engineering and cognitive psychology. It was developed to assess individuals' cognitive demands when performing complex tasks under time constraints and environmental pressure (4). One of the most widely accepted early definitions was proposed by Hart and Staveland (5), who described MWL as the perceived amount of mental effort required to complete a task, determined by the interaction between task demands, environmental conditions, and the individual's cognitive capacity and coping mechanisms (6). In the nursing context, MWL refers to the cognitive burden. This burden arises while managing simultaneous patient care responsibilities, documentation, interprofessional communication, and real-time problem-solving (7). Excessive MWL can impair attention, reduce situational awareness, and increase the likelihood of clinical errors, whereas insufficient workload may lead to disengagement and underutilization of skills (8–10). Thus, understanding MWL has become essential for optimizing staffing models, designing workflow interventions, and promoting nurse wellbeing in clinical settings.

The application of MWL in nursing research and practice has grown significantly in recent years, facilitated by validated assessment tools such as the National Aeronautics and Space Administration Task Load Index (NASA-TLX) and the Subjective Workload Assessment Technique (SWAT) (11, 12). These tools allow for quantifying cognitive strain and identifying specific workload dimensions, such as time pressure, mental demand, effort, and frustration that contribute to stress and diminished performance. However, few studies have systematically identified its multilevel predictors in real-world hospital environments. Moreover, factors such as work shift patterns, patient acuity, staffing adequacy, psychological capital, and organizational support are often studied in isolation, limiting the ability to construct a comprehensive understanding of how MWL develops and manifests in clinical practice.

In response to this gap, this study adopts a risk modeling approach grounded in the Job Demands-Resources (JD-R) theoretical framework. The aim is to explore how job demands and job or personal resources interact to shape MWL. According to the JD-R model, each profession entails unique stress-related risk factors, which are generally classified into two overarching categories: job demands and job resources (13). Job demands are defined as the physical, mental, social, or organizational characteristics of work that necessitate ongoing effort and may consequently lead to physiological or psychological strain (14). These may include time pressure, emotional labor, cognitive complexity, and role overload, all of which are prevalent in the clinical nursing environment. When job demands exceed an individual's capacity to cope, MWL increases. In contrast, job resources encompass physical, mental, social, or organizational elements that facilitate goal attainment at work, buffer the negative impact of job demands, or promote employees' personal development and growth (15). Given the multifaceted nature of mental workload, it is essential to situate the selected predictors within a coherent theoretical lens. Drawing on previous empirical findings, the variables included in this study can be meaningfully interpreted through the JD-R framework. Among them, night shift frequency and turnover intention represent typical job demands, as both increase cognitive strain, disrupt recovery, and heighten emotional and physiological stress in nursing practice (16). In contrast, salary satisfaction, emergency response capacity, and perceived social support function as job or personal resources that assist nurses in coping with demanding work conditions and mitigating cognitive overload (17). Negative coping style reflects a depletion of personal resources, which weakens individuals' ability to regulate stress effectively under high job demands (18). Gender, although not a demand or resource *per se*, has been shown to shape stress perception and emotional labor in nursing, with female nurses often reporting greater cognitive and emotional burden (19). Together, these theoretical foundations provide a structured rationale for the inclusion of the predictors and offer a clearer conceptual basis for understanding their relevance to mental workload. In this study, we propose that nurses with lower personal or social resources, such as poor coping skills, weak support networks, or insufficient emergency response capacity, are more likely to experience cognitive overload under demanding work conditions. Additionally, demographic characteristics (e.g., years of experience, job title) may function as moderators, influencing how job demands and resources interact to shape MWL.

To our knowledge, risk prediction models offer substantial benefits in nursing research and practice, especially when addressing complex psychological outcomes. In this study, a nomogram is developed as a practical and visual tool to predict high mental workload risk among clinical nurses. By integrating variables conceptualized within the D-R framework, including job demands (e.g., night shift frequency and turnover intention), job and personal resources (e.g., salary satisfaction, emergency response capacity, perceived social support, and coping style), as well as relevant demographic characteristics, the model enables personalized risk assessment, which can support nurse managers in allocating resources and designing targeted support strategies.

## 2 Methods

### 2.1 Aims

This study aimed to develop and validate a clinical prediction model for high mental workload risk among clinical nurses.

### 2.2 Study design and participants

The reporting of this study adheres to the Strengthening the Reporting of Observational Studies in Epidemiology (STROBE) guidelines for cross-sectional studies (20). A cross-sectional study was conducted across nine tertiary hospitals. Using convenience sampling, 826 registered nurses actively engaged in clinical practice were recruited between September 20 and December 31, 2024. All participating institutions were comprehensive tertiary hospitals located in Chengdu, Sichuan Province, China. These hospitals are large, high-volume medical centers that provide wide-ranging clinical services. The study sample was recruited from multiple departments, including surgical wards, medical wards, outpatient clinics, intensive care units, and operating rooms, to ensure coverage of diverse clinical settings and workload conditions. Inclusion criteria: (a) ≥1 year of clinical experience; (b) direct patient care responsibilities. Exclusion criteria: (a) administrative/educational roles only; (b) maternity/medical leave during data collection.

### 2.3 Data collection

Electronic data collection was conducted via the Questionnaire Star platform (www.wjx.cn) from September 20 to December 31, 2024. Survey links were distributed to 900 potential participants through hospital nursing administrations across nine tertiary hospitals. Encrypted electronic surveys incorporated a digital consent form preceding questionnaire access. Three automated reminders were dispatched at 72-h intervals to non-respondents. To ensure data integrity, IP address restrictions prevented duplicate submissions, and incomplete responses (< 95% items completed) were systematically excluded. From 900 returned surveys, 826 met validity criteria, yielding a final analytic sample with a 91.8% effective response rate (826/900).

### 2.4 Mental workload, candidate predictor variables and instruments

#### 2.4.1 Demographic and occupational variables

A researcher-developed questionnaire was administered to gather participants' basic demographic characteristics and job-related data. Variables included a total of 12 demographic and job-related variables, namely age, gender, marital status, educational level, years of clinical experience, department, professional title, night shift frequency, salary satisfaction, monthly income, turnover intention, and attend mental health training. These items were selected based on literature review and expert consultation to capture characteristics potentially associated with mental workload in nurses.

#### 2.4.2 Mental workload

The National Aeronautics and Space Administration Task Load Index (NASA-TLX) was employed to assess nurses' subjective mental workload (6). This widely used multidimensional tool evaluates perceived workload across six dimensions: mental demand, physical demand, temporal demand, performance, effort, and frustration. Each dimension is rated on a 21-point Likert scale ranging from 0 (very low) to 100 (very high) in increments of 5. A weighted average score is calculated to represent the overall task load, with higher scores indicating greater perceived workload. Based on the results of a systematic review and meta-analysis on the level of mental workload among nurses, the pooled mean score of mental workloads reported in the existing literature is 65.24 (21). Therefore, in the present study, a cutoff score of 65 was adopted to categorize participants into groups with or without high mental workload risk. The NASA-TLX has shown robust psychometric properties, with its reliability and validity well-established in diverse healthcare contexts, particularly within the nursing profession. In this study, the Cronbach's α was 0.773.

#### 2.4.3 Emergency response capacity

The emergency response capacity scale was originally developed by Chinese scholar Wang, to assess the emergency response capacity of community nurses in the context of public health emergencies (22). Subsequent studies have validated the scale's applicability to the broader nursing population, demonstrating good reliability and validity. The scale consists of 18 items across three dimensions. Each item is rated on a 5-point Likert scale ranging from 1 (“performed very poorly”) to 5 (“performed very well”), yielding a total score between 18 and 90. The average item score was used as the evaluation criterion: an average score < 3 indicates low emergency response capacity, a score between 3 and 4 indicates a moderate level, and a score >4 reflects a high level of emergency preparedness. In the present study, the Cronbach's α of the scale was 0.965.

#### 2.4.3 Perceived social support

The Perceived Social Support Scale, initially constructed by Zimet et al. (23) in 1983, was utilized in its Chinese adaptation translated and modified by Jiang for this study (24). The scale consists of 12 items covering three dimensions: family support, friend support, and support from others, with four items in each dimension. Responses are rated on a 7-point Likert scale, with options ranging from 1 (strongly disagree) to 7 (strongly agree), resulting in a total score that spans from 12 to 84. According to established cutoffs, scores of 12–28 indicate low perceived social support, 29–50 indicate a moderate level, and 51–84 indicate a high level. In this study, the Cronbach's α of the scale was 0.976.

#### 2.4.4 Coping style

The coping style questionnaire comprises 20 items and is a self-administered instrument developed to evaluate individuals' coping mechanisms (25). The questionnaire is structured into two subscales: positive coping style (12 items) and negative coping style (eight items). Each item is rated on a 4-point Likert scale ranging from 0 (never) to 3 (often). Higher scores in each sub-scale indicate a greater tendency to use that particular coping style. In the present study, the Cronbach's α were 0.945 for the positive coping sub-scale and 0.885 for the negative coping sub-scale.

### 2.5 Statistical analysis

Data analyses were performed using R Statistical Software (version 4.4.1). Descriptive statistics were used to summarize participants' demographic and occupational characteristics, with continuous variables presented as means ± standard deviations (SD) and categorical variables as frequencies and percentages. To identify the most relevant predictors of mental workload among nurses, the least absolute shrinkage and selection operator (LASSO) regression was conducted using the “glmnet” package to prevent overfitting and select variables with the strongest associations. In fact, LASSO regression also helps mitigate multicollinearity by shrinking the coefficients of correlated predictors, thus improving model stability. Additionally, we calculated Variance Inflation Factors (VIFs) for the variables included in the final logistic regression model to quantitatively assess multicollinearity. All VIF values were below 5, indicating no significant multicollinearity concerns. For transparency and reproducibility, categorical variables were numerically recoded before inclusion in the LASSO and binary logistic regression analyses (Table 1). Variables with non-zero coefficients in the LASSO model were retained for subsequent binary logistic regression, in which mental workload (non-high risk vs. high risk) served as the dependent variable. Adjusted odds ratios (ORs) and 95% confidence intervals (CIs) were calculated to determine the strength of associations between predictors and mental workload.

**Table 1:** Variable coding for model analyses.

**Variable**	**Type**	**Coding description**
Gender	Binary	0 = Male, 1 = Female
Marital status	Binary	0 = Married, 1 = Single
Age, years of experience	Continuous	Original numeric values
Education level	Ordinal	0 = Associate or below, 1 = Bachelor's, 2 = Master's or above
Years of nursing experience	Ordinal	0 = 1–10 years, 1 = 11–20 years, 2 = >20 years
Professional title	Ordinal	0 = Staff nurse, 1 = Senior staff nurse, 2 = Advanced practice nurse
Department	Ordinal	0 = Internal Medicine, 1 = Surgery, 2 = Critical Care/Emergency Units, 3 = Other Departments
Salary satisfaction	Ordinal	0 = Dissatisfied, 1 = Slightly unsatisfied, 2 = Satisfied, 3 = Very satisfied
Monthly income	Ordinal	0 = ≤ 5,000RMB, 1 = 5,001–8,000RMB, 2 = 8,001–10,000RMB, 3 = >10,000RMB
Night shift frequency	Ordinal	0 = 0–3/Month, 1 = 4–6/Month, 2 = >6/Month
Turnover intention	Ordinal	0 = Never, 1 = Rarely, 2 = Occasionally, 3 = Often
Attend mental health training	Binary	0 = Yes, 1 = No
Emergency response capacity	Ordinal	0 = Low, 1 = Moderate, 2 = High
Perceived social support	Ordinal	0 = Low, 1 = Moderate, 2 = High
Positive/Negative coping style	Continuous	Entered as continuous scale scores

A nomogram was then developed based on the final logistic regression model to provide a visual and practical tool for individualized risk estimation. The model's discriminative performance was evaluated by the area under the receiver operating characteristic curve (AUC), with values approaching 1.0 indicating superior predictive accuracy. Model calibration was examined through calibration plots alongside the Hosmer–Lemeshow goodness-of-fit test. To assess stability and generalizability, internal validation was performed via 10-fold cross-validation. Statistical significance was determined at a two-tailed *P*-value threshold of less than 0.05.

### 2.6 Ethical considerations

All participating nurses gave informed consent and were made aware of their right to withdraw from the study at any time without repercussions. Ethical approval for this study was obtained from the Medical Ethics Committee, Affiliated Hospital of Chengdu University of Traditional Chinese Medicine (Number: 2023KL-098; Date: July 31, 2023).

## 3 Results

### 3.1 Demographics and prevalence of mental workload among nurses

A total of 826 clinical nurses participated in this study. Among them, participants included 177 males (21%) and 649 females (79%), mean age was 30.52 years (SD = 8.87). Most nurses were married, staff nurse and had a nursing education of bachelor's degree. Only 15% of nurses had >20 years of nursing experience; 30% had never considered leaving their position. 330 nurses had a total mean mental workload score < 65, accounting for approximately 40% of the total number. 496 nurses had a mental workload score ≥65, accounting for approximately 60% of the total. So, approximately 60% of clinical nurses were at risk of high mental workload. The comparison of variable characteristics between different mental workload was shown in Table 2. Significant differences were observed in gender, salary satisfaction, frequency of night shifts, turnover intention, emergency response capacity, perceived social support, positive coping style and negative coping style between the high mental workload Group and non-high mental workload Group (*P* < 0.05).

**Table 2:** Comparison of variable characteristics between different mental workload.

**Variables**	**Category**	**Mental workload**			***P-*value**	**Statistic**
		**Total (*****n*** = **826)**	**Non-high risk (*****n*** = **330)**	**High risk (496)**		
Gender (%)	Male	177 (21)	112 (34)	65 (13)	**< 0.001**	49.859
	Female	649 (79)	218 (66)	431 (87)		
Age (years, Mean, SD)		30.52 ± 8.87	30.98 ± 9.03	30.21 ± 8.76	0.222	1.232
Marital status (%)	Marriage	426 (52)	156 (47)	270 (54)	0.052	3.789
	Single	400 (48)	174 (53)	226 (46)		
Level of education (%)	Associate degree or less	292 (35)	125 (38)	167 (34)	0.224	2.989
	Bachelor's degree	470 (57)	176 (53)	294 (59)		
	Master's degree or above	64 (8)	29 (9)	35 (7)		
Years of nursing experience (%)	1–10 years	553 (67)	212 (64)	341 (69)	0.323	2.258
	11–20 years	145 (18)	60 (18)	85 (17)		
	>20 years	128 (15)	58 (18)	70 (14)		
Professional title (%)	Staff nurse	383 (46)	146 (44)	237 (48)	0.463	1.541
	Senior staff nurse	288 (35)	116 (35)	172 (35)		
	Advanced practice nurse	155 (19)	68 (21)	87 (18)		
Department (%)	Internal medicine	154 (19)	66 (20)	88 (18)	0.694	1.450
	Surgery	548 (66)	213 (65)	335 (68)		
	Critical care/Emergency units	51 (6)	23 (7)	28 (6)		
	Other departments	73 (9)	28 (8)	45 (9)		
Salary satisfaction (%)	Dissatisfied	321 (39)	86 (26)	235 (47)	**< 0.001**	92.657
	A little unsatisfied	204 (25)	71 (22)	133 (27)		
	Satisfied	163 (20)	71 (22)	92 (19)		
	Very satisfied	138 (17)	102 (31)	36 (7)		
Monthly income (RMB, %)	≤ 5,000	220 (27)	79 (24)	141 (28)	0.512	2.304
	5,001–8,000	194 (23)	80 (24)	114 (23)		
	8,001–10,000	194 (23)	78 (24)	116 (23)		
	>10,000	218 (26)				
Night shifts frequency (Month, %)	0–3	348 (42)	155 (47)	193 (39)	**0.001**	13.762
	4–6	306 (37)	127 (38)	179 (36)		
	>6	172 (21)	48 (15)	124 (25)		
Turnover intention (%)	Never considered	164 (20)	96 (29)	68 (14)	**< 0.001**	32.219
	Rarely considered	248 (30)	78 (24)	170 (34)		
	Occasionally considered	219 (27)	85 (26)	134 (27)		
	Often considered	195 (24)	71 (22)	124 (25)		
Attend mental health training (%)	Yes	502 (61)	212 (64)	290 (58)	0.111	2.535
	No	324 (39)	118 (36)	206 (42)		
		**Total (*****n*** = **826)**	**Non-high risk (*****n*** = **330)**	**High risk (496)**		
Emergency response capacity (%)	Low level	169 (20)	36 (11)	133 (27)	**< 0.001**	51.416
	Moderate level	481 (58)	190 (58)	291 (59)		
	High level	176 (21)	104 (32)	72 (15)		
Perceived Social support (%)	Low level	46 (6)	3 (1)	43 (9)	**< 0.001**	40.463
	Moderate level	240 (29)	74 (22)	166 (33)		
	High level	540 (65)	253 (77)	287 (58)		
Positive coping style (Mean, SD)		20.22 ± 7.84	21.53 ± 8.46	19.34 ± 7.27	**< 0.001**	3.951
Negative coping style (Mean, SD)		14.67 ± 5.44	13.02 ± 5.54	15.77 ± 5.09	**< 0.001**	7.338

Bold values indicate statistically significant differences (*p* < 0.05).

### 3.2 Variable selection

**Figure 1:**
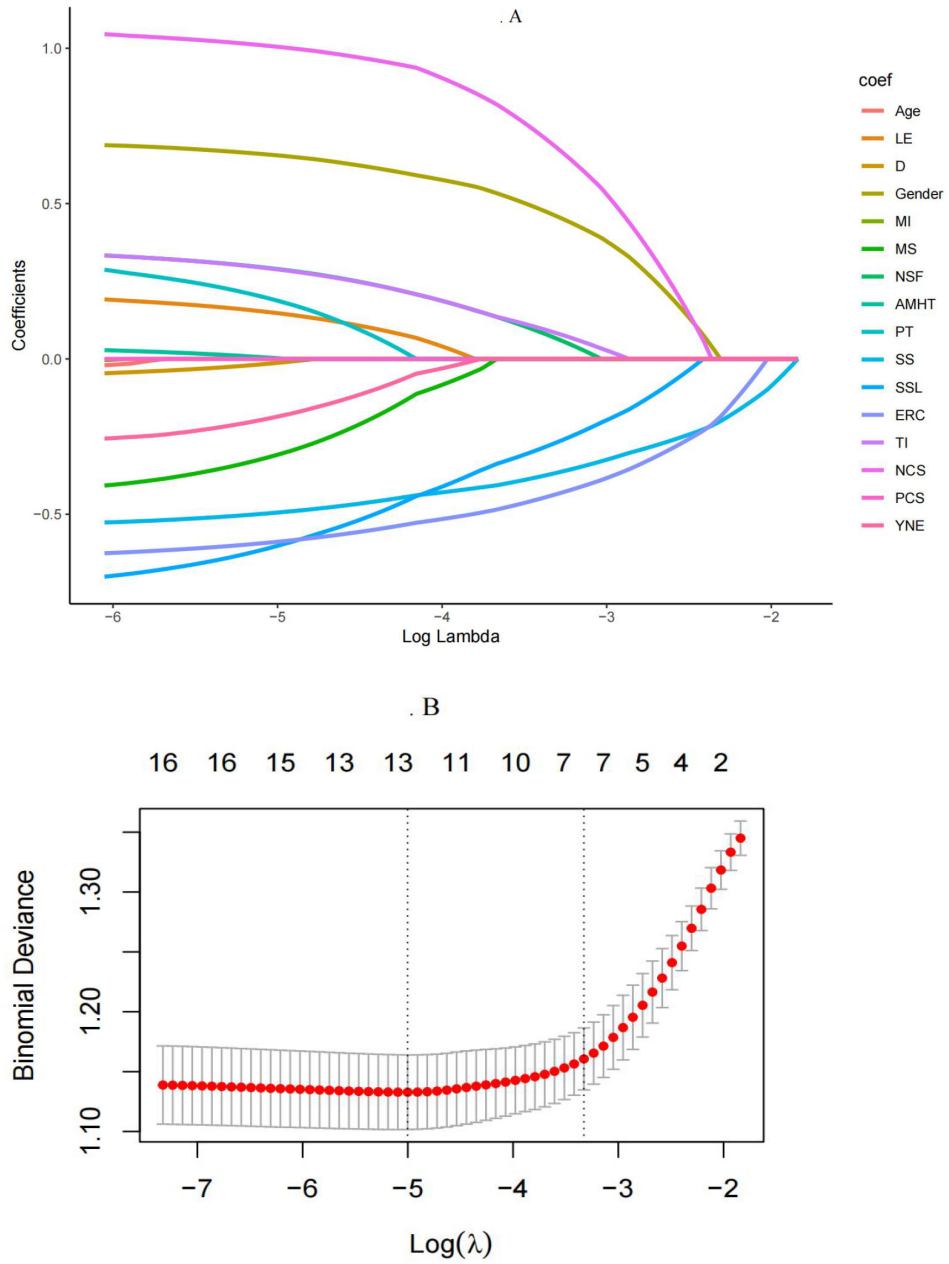
Variable selection process using LASSO regression. **(A)** LASSO coefficient profiles of the 16 baseline features; **(B)** Tuning parameter (λ) selection in the LASSO model used 20-fold cross-testing via minimum criteria. LE, level of education; D, department; MI, monthly income; MS, marital status; NSF, night shifts frequency; AMHT, attend mental health training; PT, professional title; SS, Salary satisfaction; SSL, social support level; ERC, Emergency response capacity; TI, turnover intention; NCS, negative coping style; PCS, positive coping style; YNE, years of nursing experience. Cohort size: total *n* = 826.

A total of 16 indicators were incorporated into the LASSO regression analysis. As illustrated in Figures 1A, B, the analysis identified gender, salary satisfaction, frequency of night shifts, turnover intention, emergency response capacity, perceived social support and negative coping style are significant predictive factors for high mental workload prevalence among nurses.

These seven variables were incorporated into the binary logistic regression analysis, which revealed that the gender (female; OR, 2.256, 95% CI: 1.403–3.652; *P* < 0.001), salary satisfaction(satisfied, very satisfied; OR, 0.506, 95% CI: 0.288–0.886; *P* < 0.05; OR, 0.156, 95% CI: 0.085–0.279, *P* < 0.001), frequency of night shifts (>6 times per month; OR, 2.124, 95% CI: 1.22–3.764, *P* < 0.01), turnover intention (rarely considered: OR, 2.763, 95% CI:1.611–4.791; *P* < 0.001; occasionally considered: OR, 2.049, 95% CI:1.138–3.721, *P* < 0.05; often considered: OR, 2.651, 95% CI:1.465–4.87, *P* < 0.01), emergency response capacity(moderate level: OR, 0.465, 95% CI:0.255–0.819; *P* < 0.01; high level: OR, 0.258, 95% CI:0.127–0.507; *P* < 0.001), Social support (moderate level: OR, 0.175, 95% CI:0.026–0.667; *P* < 0.05; high level: OR, 0.136, 95% CI: 0.021–0.503; *P* < 0.01), and negative coping style (OR, 1.053, 95% CI:1.015–1.093, *P* < 0.05). Table 3 were significant independent predictors of mental workload among nurses.

**Table 3:** Binary logistic regression analysis of mental workload among nurses.

**Predictors**	**β**	**SE**	**Wald**	**OR (95% CI)**	***P*-value**
(Intercept)	1.434	0.941	2.321	4.194 (0.764–34.531)	0.127
**Gender**					
Male	Ref (–)				–
Female	0.814	0.244	11.153	2.256 (1.403–3.652)	**< 0.001**
**Salary satisfaction**					
Dissatisfied	Ref (–)				–
A little unsatisfied	−0.467	0.269	3.014	0.627 (0.37–1.063)	0.082
Satisfied	−0.682	0.286	5.678	0.506 (0.288–0.886)	**< 0.05**
Very satisfied	−1.859	0.303	37.669	0.156 (0.085–0.279)	**< 0.001**
**Night shifts frequency**					
0–3	Ref (–)				–
4–6	−0.070	0.222	0.101	0.932 (0.602–1.44)	0.751
>6	0.753	0.287	6.897	2.124 (1.22–3.764)	**< 0.01**
**Turnover intention**					
Never considered	Ref (–)				–
Rarely considered	1.016	0.278	13.401	2.763 (1.611–4.791)	**< 0.001**
Occasionally considered	0.717	0.302	5.652	2.049 (1.138–3.721)	**< 0.05**
Often considered	0.975	0.306	10.159	2.651 (1.465–4.87)	**< 0.01**
**Emergency response capacity**					
Low level	Ref (–)				–
Moderate level	−0.765	0.296	6.680	0.465 (0.255–0.819)	**< 0.01**
High level	−1.357	0.352	14.889	0.258 (0.127–0.507)	**< 0.001**
**Perceived Social support**					
Low level	Ref (–)				–
Moderate level	−1.742	0.784	4.937	0.175 (0.026–0.667)	**< 0.05**
High level	−1.995	0.773	6.661	0.136 (0.021–0.503)	**< 0.01**
Negative coping style	0.052	0.019	7.325	1.053 (1.015–1.093)	**< 0.01**

Bold values indicate statistically significant differences (*p* < 0.05).

### 3.3 Construction of the predictive model of mental workload

**Figure 2:**
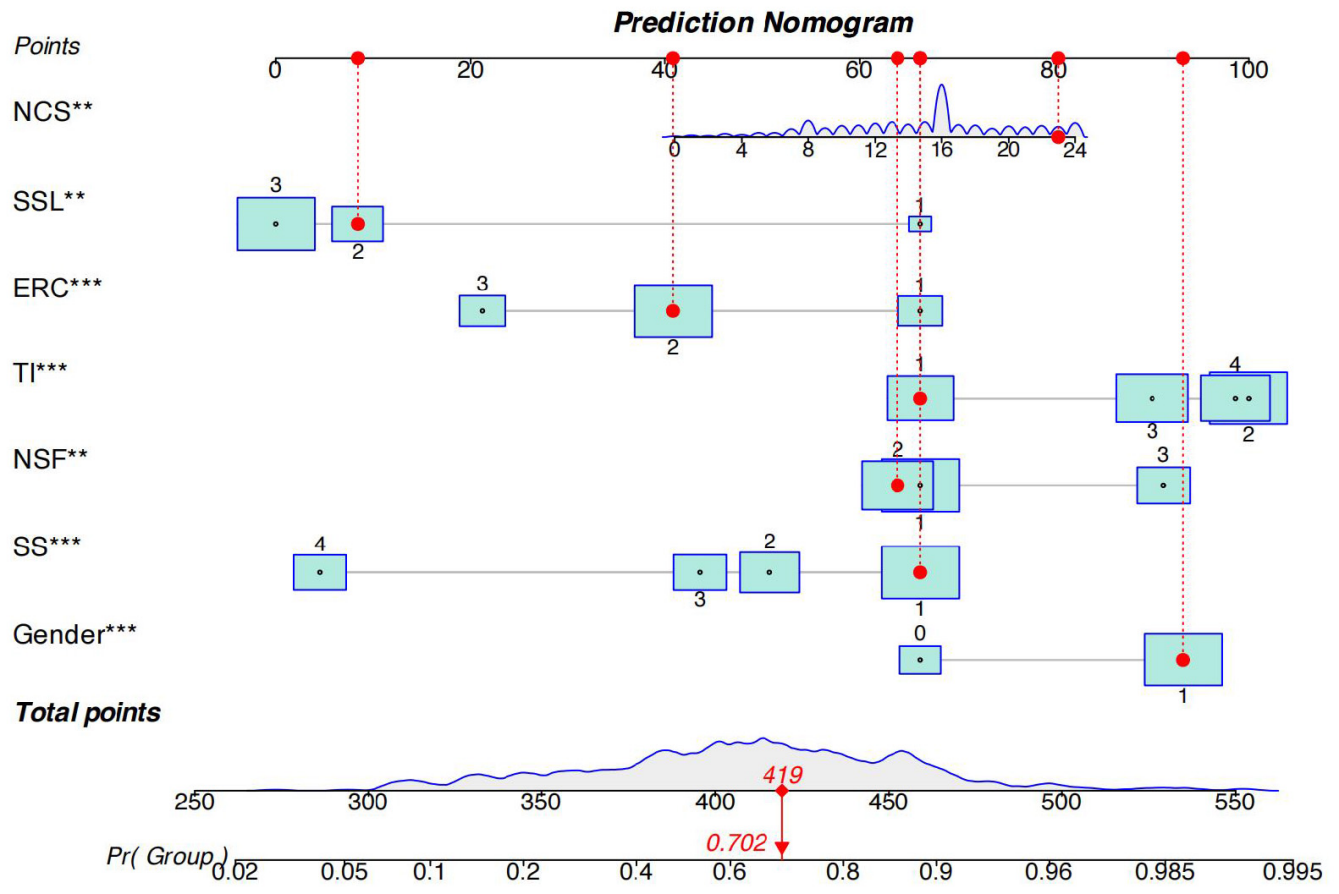
Nomogram predicting the probability of high mental workload among nurses (training cohort, *n* = 578). Each predictor is assigned a specific point value; total scores correspond to predicted probabilities of high mental workload. NCS, negative coping style; SSL, social support level; ERC, Emergency response capacity; TI, turnover intention; NSF, night shifts frequency; SS, Salary satisfaction.

A nomogram was developed to predict the risk of high mental workload using the training cohort (70% of the total sample, *n* = 578), and its performance was subsequently validated in the internal validation cohort (30% of the total sample, *n* = 248). Drawing on the results from the binary logistic regression analysis, seven variables were incorporated into the nomogram (Figure 2). This nomogram translates each variable's value into a specific point score. The cumulative score corresponds vertically to the predicted probability of elevated mental workload among nurses. A higher total score indicates an increased risk of experiencing high mental workload. The optimal cut off for predicting high mental workload prevalence in the nomogram is 419 points. So, the nurses had a 70.2 % risk of experiencing high mental workload by the nomogram model.

### 3.4 Evaluation of the predictive model of mental workload

**Figure 3:**
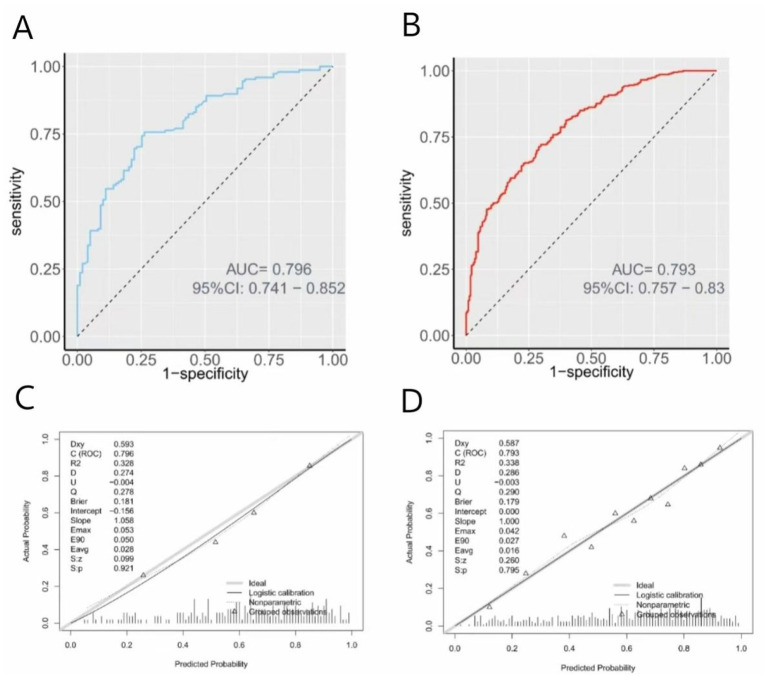
ROC curve and calibration curve for the nomogram based on the training cohort and validation cohort. **(A, C)** training cohort (*n* = 578); **(B, D)** validation cohort (*n* = 248). ROC, receiver operating characteristic; AUC, area under the curve.

In both the training and validation cohorts, the nomogram demonstrated strong discriminative capacity in distinguishing clinical nurses with high mental workload from those without, yielding AUC values of 0.796 (95% CI: 0.741–0.852) and 0.793 (95% CI: 0.757–0.830), respectively (Figures 3A, B). The calibration curves indicated good agreement between the predicted and observed probabilities, supported by Hosmer–Lemeshow test *P*-values of 0.921 and 0.795 for the training and validation sets, respectively (Figures 3C, D).

**Figure 4:**
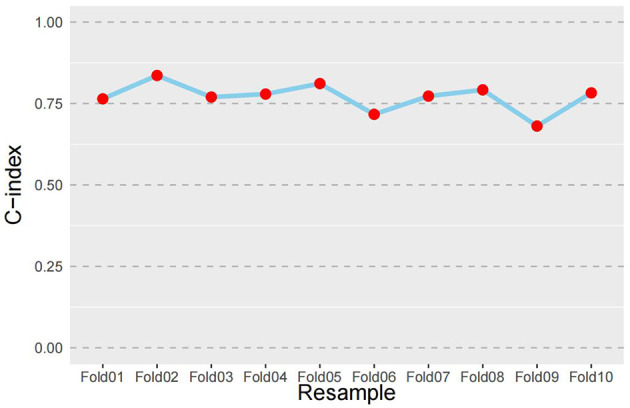
Ten-Fold Cross-Validation performance of the mental workload risk prediction nomogram (*n* = 826). Blue lines indicate training folds; red markers indicate test folds; C-index: concordance index.

Furthermore, the predictive performance of the nomogram was rigorously evaluated through 10-fold cross-validation. As depicted in Figure 4, the dataset was randomly partitioned into 10 equally sized subsets. During iterative validation cycles, each fold served as an independent test set (red markers) while the remaining 90% formed the training set (blue lines). Logistic regression models were rebuilt for each training partition, with discrimination performance evaluated via C-index on corresponding test sets. Bootstrap resampling (1,000 iterations) generated a C-index of 0.761 (95% CI: 0.709–0.814), while 10-fold cross-validation produced a mean C-index of 0.771. This close agreement (difference = 0.01) indicates consistent predictive accuracy across validation frameworks. Both values substantially exceed the chance threshold of 0.5, confirming the model's clinical utility for mental workload risk stratification.

## 4 Discussion

### 4.1 The prevalence of high mental workload among nurses

Mental workload is a persistent health issue experienced by clinical nurses over the long term (32). A high level of mental workload negatively affects nurses' mental health, the quality of patient care, and overall organizational development (28, 29). In the present survey, approximately 60% of clinical nurses were found to be experiencing a high level of mental workload, indicating that a substantial proportion of the nursing workforce was subjected to sustained cognitive and emotional demands in their daily practice. The prevalence of this issue is high and may lead to negative consequences, such as emotional exhaustion and decreased job satisfaction among nurses, as well as a decline in the quality of patient care. Therefore, targeted strategies are urgently needed to monitor and alleviate nurses' mental workload. Furthermore, the proportion of individuals in our study who experienced a high level of mental workload was close to, or consistent with, the proportion reported in an earlier study (21, 30). This suggested that the issue of high mental workload in nurses was persistent or widely observed across different settings or populations. Therefore, it is essential for hospital administrators to promptly identify nurses at high risk of experiencing elevated mental workload and to pay close attention to their coping mechanisms in order to mitigate the potential negative impact and prevent further psychological strain.

### 4.2 Predictors of mental workload among clinical nurses

This study identified seven significant predictors of high mental workload among nurses: gender, salary satisfaction, frequency of night shifts, turnover intention, emergency response capacity, perceived social support, and negative coping style. According to the JD-R model, these predictors can be categorized into two major groups. Specifically, frequency of night shifts and turnover intention represent job demands that heighten nurses' cognitive and emotional strain. In contrast, salary satisfaction, emergency response capacity, and perceived social support serve as job or personal resources that buffer the adverse effects of job demands and foster resilience. Negative coping style reflects a deficit of personal resources, which amplifies stress reactions under high workload conditions. It is noted that negative coping style was significantly associated with mental workload in this study. However, its relatively small effect size suggests that it serves as one of several subtle contributors rather than a primary driver. This finding is consistent with the broader understanding that coping strategies influence individuals' stress perception and regulation, often in interaction with other psychological and environmental factors. Clinically, even modest increases in mental workload related to negative coping may accumulate over time or exacerbate the effects of other stressors. This highlights that the importance of promoting adaptive coping skills as part of comprehensive interventions to support nurses' wellbeing. While gender functions as an individual characteristic that may moderate the interplay between job demands, resources, and perceived mental workload. Our findings revealed that female nurses were more than twice as likely to experience high mental workload compared to males. This pattern has been reported in prior nursing and occupational health studies. It is thought to reflect differences in role expectations, emotional job demands, and organizational environments in which women are more frequently placed. Evidence indicates that female nurses tend to undertake a greater share of emotional labor, assume more patient-facing responsibilities, and face heightened expectations regarding communication and relational work compared with male nurses (33). Gendered task allocation and unequal access to informal workplace support have also been noted as contributors to higher perceived workload among women (35). These differences increase both emotional and cognitive effort during daily tasks. However, the gender imbalance in our sample, with substantially fewer male participants, may limit the robustness of this finding. Future studies with more balanced gender distributions, preferably through multicenter or stratified sampling, are needed to further verify this association and clarify the mechanisms underlying gender-related differences in mental workload.

Consistent with the JD-R model, when job demands exceed coping capacity, strain increases, leading to higher perceived workload. Salary satisfaction, acting as a key job resource, served as a protective factor against high mental workload. Nurses who were satisfied with their income perceived greater organizational support and motivation, which buffered the adverse impact of heavy demands (36). Similarly, frequent night shifts constitute a demanding work condition that disrupts circadian rhythms and recovery processes, thereby increasing fatigue and emotional exhaustion (31). Turnover intention was another strong predictor of high mental workload. Nurses who reported occasionally or frequently considering leaving their job were significantly more likely to experience excessive workload. This finding highlighted the reciprocal relationship between mental strain and turnover intention, as heavy workload could both result from and contribute to job dissatisfaction and disengagement (37).

Regarding psychological and social resources, emergency response capacity and perceived social support emerged as significant protective factors, consistent with earlier studies (18, 38). Higher emergency preparedness enhances nurses' self-efficacy, enabling them to manage clinical stressors more effectively, whereas strong social support provides emotional and instrumental resources that mitigate the impact of job demands. These resources help maintain engagement and effective coping despite high workloads, in line with the motivational pathway of the JD-R model. Finally, a negative coping style functions as a personal resource deficit, reducing resilience and increasing vulnerability to stress. This underscores the importance of fostering positive coping strategies and strengthening both organizational and personal resources to promote nurses' wellbeing and sustain high-quality patient care (26). Interventions should therefore aim to reduce excessive job demands while simultaneously enhancing organizational and personal resources to promote nurses' wellbeing and sustain high-quality patient care.

### 4.3 Construction and clinical application of the predictive model for mental workload among nurses

In this study, we developed a nomogram-based model to estimate the probability of high mental workload among clinical nurses. The model incorporates seven independent predictors and converts each into a weighted point value. The sum of these values produces an individualized total score, which reflects the nurse's overall risk (27). A score above 419 points, corresponding to a predicted probability of 70.2%, was identified as the optimal cutoff for determining high mental workload. This threshold provides a reasonable balance between sensitivity and specificity. It allows the model to identify most nurses who are experiencing substantial cognitive strain, while still distinguishing those with lower workload levels. In practice, false-positive results may lead to offering additional support to individuals who are not severely overloaded, but such interventions pose minimal risk. In contrast, false-negative cases may leave high-risk nurses unidentified, which could contribute to ongoing mental workload and reduced performance. Therefore, the chosen cutoff emphasizes early detection and prevention. In clinical settings, the nomogram can be applied as part of routine nursing management. All included predictors, such as night shift frequency, turnover intention, emergency response capacity, and perceived social support are easy to obtain. By entering these data, managers can quickly identify individuals whose workload risk exceeds the recommended threshold. These nurses can then be considered for targeted interventions, including workload adjustments, psychological support, or training in stress-management skills. Moreover, the model can be incorporated into digital nursing management systems to guide shift scheduling, identify individuals who are more vulnerable to cognitive overload, and ensure a more balanced distribution of work demands across units.

In terms of discriminative ability, the model achieved strong area under the curve (AUC) values of 0.796 in the training cohort and 0.793 in the validation cohort. These results indicate robust classification performance, surpassing the commonly accepted threshold of 0.70 for good discrimination (34). The calibration analysis, supported by high Hosmer–Lemeshow test *P*-values (0.921 and 0.795), further confirmed that the predicted probabilities closely aligned with actual outcomes, indicating excellent model reliability and calibration. To ensure robustness and internal validity, the model was also subjected to 10-fold cross-validation and bootstrap resampling (1,000 iterations). The resulting C-indices from cross-validation (0.771) and bootstrap resampling (0.761) showed a minimal difference of 0.01, highlighting the model's robustness, stability, and reproducibility across different data subsets. These results collectively confirmed that the nomogram was not overfitted to the training data and is likely to maintain good performance in similar clinical settings. From a theoretical standpoint, the model is grounded in the Job Demands-Resources (JD-R) model, incorporating both job demands (e.g., night shifts, turnover intention) and job/personal resources (e.g., emergency response capacity, social support, coping style). This integration enhances the nomogram's conceptual validity and provides a holistic view of mental workload risk rooted in occupational health theory.

## 5 Limitations

This study has several limitations that should be acknowledged. First, the cross-sectional nature of the survey limits the ability to establish causal relationships between the predictors and mental workload. Second, as both predictors and outcomes were assessed using self-reported measures, the findings may be influenced by common method bias, potentially inflating the strength of the observed associations and the model's predictive accuracy. Furthermore, the model relies solely on subjective psychological and occupational variables without incorporating objective or physiological indicators of workload, which may restrict the comprehensiveness of the construct measured. Third, the NASA-TLX cutoff score of 65 was adopted from a result of meta-analysis may not reflect our local sample accurately. In the absence of local pilot data and sensitivity analyses, this threshold remains provisional and requires further validation. Fourth, although the nomogram demonstrated strong performance in internal validation, including bootstrap resampling and 10-fold cross-validation, the absence of external validation remains a significant limitation. The model's generalizability to other hospitals, regions, and clinical environments is unknown. Finally, the use of convenience sampling may limit the representativeness of the sample. Although the participating institutions are all large tertiary hospitals, the sampling method may introduce selection bias and restrict the generalizability of our findings to other regions, hospital tiers, or care models. Future research should prioritize multicenter external validation and prospective evaluation to assess the stability of predictive accuracy across diverse populations and organizational contexts. Additionally, it should validate workload thresholds using local data, incorporate objective measures, and employ probability sampling across different regions and hospital types to enhance robustness and applicability.

## 6 Conclusions

This study developed and internally validated a predictive nomogram for assessing the risk of high mental workload among clinical nurses. The model incorporated seven key predictors: gender, salary satisfaction, night shift frequency, turnover intention, emergency response capacity, perceived social support, and negative coping style, and demonstrated strong discrimination, calibration, and stability through bootstrap resampling and cross-validation. Rooted in the Job Demands-Resources (JD-R) framework, the model synthesizes both job-related demands and psychosocial resources, offering a holistic and theory-grounded approach to assessing mental workload. By providing an interpretable and quantifiable risk estimation tool, this nomogram can assist nurse managers in identifying at-risk individuals early and implementing personalized preventive strategies. The integration of modifiable factors, such as coping style and social support, also highlights potential entry points for targeted intervention programs. Future research should focus on external validation and longitudinal evaluation to further establish the tool' s practical relevance and generalizability across healthcare settings.

## Data Availability

The original contributions presented in the study are included in the article/supplementary material, further inquiries can be directed to the corresponding author.
